# A case report of refractory otalgia after Ramsay Hunt syndrome successfully treated by applying pulsed radiofrequency to the great auricular nerve: A CARE-compliant article

**DOI:** 10.1097/MD.0000000000027285

**Published:** 2021-10-01

**Authors:** Ye Sull Kim, Ji-Seon Son, Hyungseok Lee, A. Ram Doo

**Affiliations:** a Department of Anesthesiology and Pain Medicine, Jeonbuk National University Hospital and Medical School, Jeonju, South Korea; b Research Institute of Clinical Medicine of Jeonbuk National University-Biomedical Research Institute of Jeonbuk National University Hospital, Jeonju, South Korea.

**Keywords:** great auricular nerve, postherpetic neuralgia, radiofrequency, Ramsay Hunt syndrome

## Abstract

**Rationale:**

Ramsay Hunt syndrome is a type of herpes zoster infection involving geniculate ganglion and facial nerve. Unilateral facial palsy, otalgia, and painful vesicular rash on the auricle and external auditory canal are the typical symptoms. Although postherpetic neuralgia (PHN) is a devastating complication of herpes zoster infection, PHN following Ramsay Hunt syndrome has rarely been reported.

**Patient concerns:**

A 55-year-old immunocompetent female patient visited our pain clinic, for left-sided refractory otalgia (PHN) that persisted for 3 months after she was diagnosed with Ramsay Hunt syndrome. Although facial palsy and tinnitus had recovered within 2 to 4 weeks after symptom onset, the patient had been experiencing a persistent and severe otalgia radiating to mandibular angle, temporal and upper cervical area of neuropathic nature.

**Diagnoses:**

The patient's pain persisted despite conservative medication and administration of ultrasound-guided stellate ganglion block, facial nerve block, and great auricular nerve block several times.

**Interventions:**

The patient was treated with the application of ultrasound-guided pulsed radiofrequency (PRF) to the great auricular nerve.

**Outcomes:**

The patient experienced significant pain reduction more than 50% on a numeric rating scale after 2 weeks of PRF treatment.

**Lessons:**

Chronic otalgia might be a type of PHN after Ramsay Hunt syndrome with cervical nerve involvement. PRF treatment to the great auricular nerve can be a therapeutic option for refractory otalgia following Ramsay Hunt syndrome.

## 1 Introduction

Ramsay Hunt syndrome is a rare type of herpes zoster infection involving the 7th cranial nerve, that is, facial nerve.^[1]^ Reactivation of the varicella zoster virus, which remains dormant in the geniculate ganglion is responsible for this rare neurological disorder. Unilateral facial palsy, otalgia, and painful vesicular rash on the auricle and external auditory canal are the typical symptoms. Moreover, vesicles may occur in the oral mucosa, along the distribution of the facial nerve.^[2]^ Ramsay Hunt syndrome is a self-limiting viral disease, but facial palsy may be the primary morbidity in some patients.^[3]^ Although postherpetic neuralgia (PHN) is a devastating complication of herpes zoster infection, PHN following Ramsay Hunt syndrome has rarely been reported.

In this case report, we encountered a female patient who was diagnosed with Ramsay Hunt syndrome 3 months ago. She had been experiencing persistent and severe otalgia radiating to mandibular angle, temporal and upper cervical area of neuropathic nature. Here, we present the successful treatment of chronic otalgia (i.e., PHN) after Ramsay Hunt syndrome using pulsed radiofrequency (PRF) to the great auricular nerve.

## 2 Case report

A 55-year-old immunocompetent female patient visited our pain clinic for persisting left-sided chronic otalgia 3 months after being diagnosed with Ramsay Hunt syndrome. The patient complained of continuous dull pain in the external auditory meatus and an earlobe, of a numeric rating of 4 on an 11-point numeric rating scale (NRS), and a sharp and shooting pain, sometimes radiating to the mandibular angle, temporal and upper cervical area, with a numeric rating of 9/10 during a pain attack.

Three months prior to visiting our center, the patient had presented to a primary care clinic with acute-onset left-sided facial palsy and otalgia with vesicular formation on the auricle. Subsequently, she experienced dizziness, tinnitus, and aural fullness. The patient had unilateral hypoesthesia of the face and a horizontal spontaneous right-beating nystagmus. Although she had no chronic or immunological disease, she had experienced a rib fracture due to a car accident 2 weeks prior. After symptom onset, she received intravenous antiviral treatment (acyclovir 1200 mg daily) for 7 days in the primary care clinic, and she was referred to our tertiary care hospital for further treatment.

Pure tone audiometry report indicated that she had a high-tone sensorineural hearing loss averaging 25 dB. Caloric test findings showed left-sided weakness (45.11%) and rightward preponderance. Magnetic resonance imaging revealed left facial nerve neuritis with enhancement, compatible with Ramsay Hunt syndrome. Vascular compression of cranial nerves, including the trigeminal, facial, glossopharyngeal, and vagus nerves, was not observed. The patient was diagnosed with Ramsay Hunt syndrome complicated by multiple cranial nerve involvement (cranial nerves V and VIII). Conservative treatment, including steroid pulse treatment and vestibular rehabilitation therapy, was administered. Methylprednisolone (50 mg daily) was administered intravenously for 7 days. Her initial symptoms, including dizziness, tinnitus, and facial palsy, recovered nearly 2 to 4 weeks after onset; however, severe otalgia persisted for more than 3 months, and she was referred to our pain clinic for further treatment.

After reporting to our pain clinic, the patient was administered medications, including pregabalin and nortriptyline, with incremental doses up to 300 mg/day and 50 mg/day, respectively, and analgesics, such as tramadol/paracetamol combination 75/650 mg daily or tapentadol 100 mg/day. Ultrasound-guided facial nerve and stellate ganglion block was performed every 2 weeks. However, the pain-relieving effect of the procedure was transient only for 3 to 4 days, and the patient's symptoms did not improve (continuous pain and shooting pain of NRS 4 and 7, respectively), even with dose increment of medications and repeated nerve blocks. Because she also presented radiating pain along the cutaneous distribution of great auricular nerve, ultrasound-guided great auricular nerve block was added. It also showed transient symptomatic relief. Consequently, the application of PRF to the great auricular nerve was planned.

**Figure 1:**
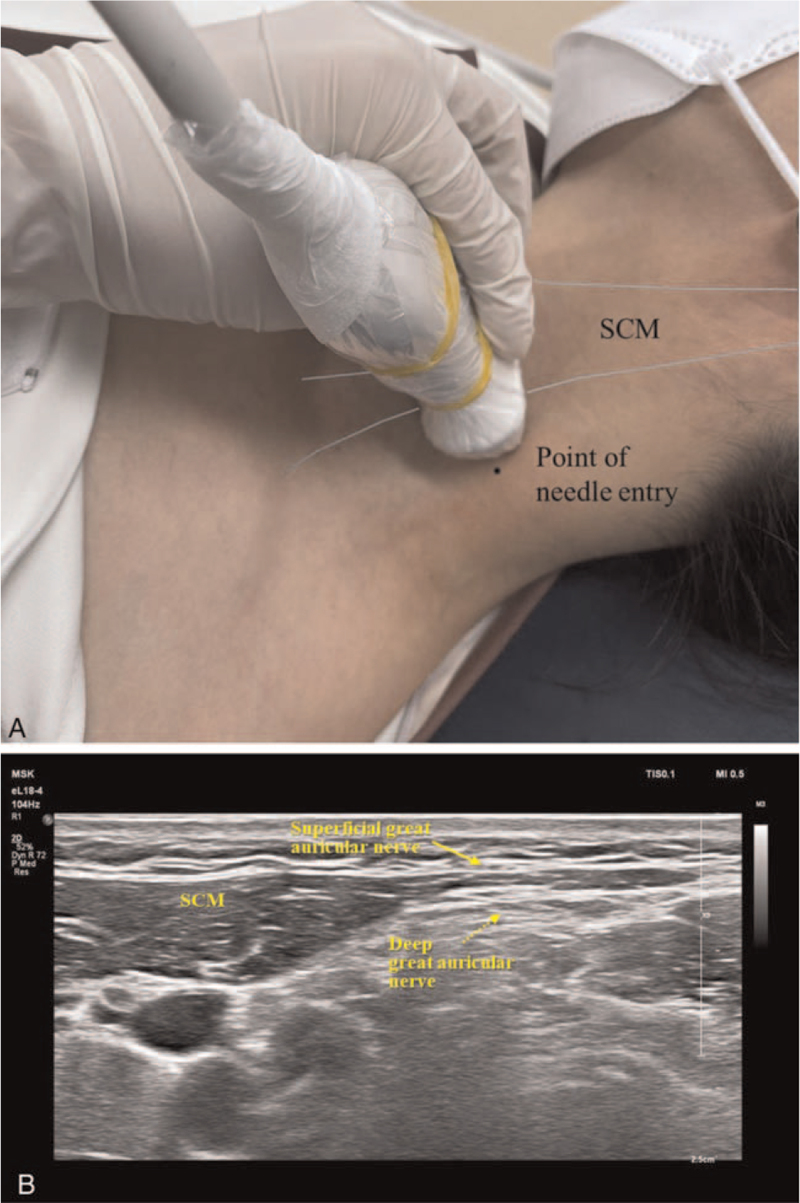
Ultrasound scanning of the great auricular nerve. (A) Placement of probe for optimal visualizing of the great auricular nerve. (B) Transverse-oblique sonographic view of both superficial (arrow) and deep (dotted arrow) great auricular nerve. SCM = sternocleidomastoid muscle.

**Figure 2:**
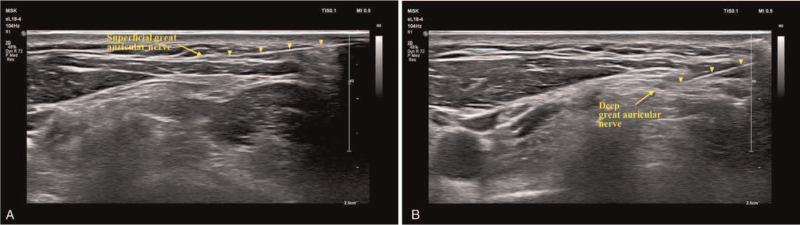
Pulsed radiofrequency (PRF) needle (arrow head) reaching the great auricular nerve under real-time ultrasonography. (A) PRF application to the superficial great auricular nerve. (B) PRF application to the deep great auricular nerve.

The patient was placed in supine position with her head tilt to the opposite site. The posterior border of the sternocleidomastoid (SCM) muscles at the cricoid cartilage level was scanned with a 18 to 4 MHz linear array transducer (Affinity 70, Philips Ultrasound, Bothell, WA) in a transverse-oblique view (Fig. 1A). After identifying superficial cervical plexus lying posterior to the midpoint of the SCM, the great auricular nerve was identified by scanning with a cephalad direction as it arose from superficial cervical plexus. It curved around the SCM muscle as moving superficially in an anteromedial direction to lie on the anterior surface of SCM. In this portion, both superficial and deep great auricular nerve are well visualized (Fig. 1B). After usual sterilization and skin infiltration, a 22-guage, 5-cm insulated needle with a 5-mm active tip (Abbott Medical, Plymouth, MN) was inserted toward the superficial and deep great auricular nerve with a posterior to anterior direction under real-time ultrasonography using in-plane technique (Fig. 2A and B). When the needle reached the great auricular nerve, electrical stimulation was performed. The needle position was carefully adjusted to achieve paresthesia along with the nerve territory at 0.5 V with 50 Hz sensory stimulation. The patient reported appropriate paresthesia on the auricle, external auditory meatus, and mandibular angle where she had been experiencing pain previously. Thereafter, PRF treatment at 42°C, 45 V for 360 seconds, with a pulse frequency of 2 Hz and a pulse width of 20 ms, was performed using RF generator (Cosman G4, Cosman Medical, Burlington, MA). After modifying the needle position slightly with the same process as described above, a cycle of PRF was repeated. The procedure was well tolerated by the patient and was completed without any complications.

At 2-weeks follow-up, the patient reported remarkable and satisfactory pain reduction of 50%. She reported that the intensity of the shooting pain was decreased from 9 to 4 on the NRS, and the frequency of pain also decreased. Continuous dull pain (resting pain) was also decreased from 4 to 2 of NRS. The patient reported no complications after the procedure.

## 3 Discussion

In 1968, Ramsay Hunt first described a rare herpetic inflammation of the geniculate ganglion with erythematous vesicle on ear or oral mucosa, known as herpes zoster oticus or Ramsay Hunt syndrome.^[4]^ It may manifest a variety of clinical symptoms because of anatomical complexity and broad function of facial nerve. And it might involve multiple cranial nerve, which is herpes zoster-associated cranial polyneuropathy.^[5]^ Concomitant involvement of the vestibulocochlear (CN VIII) or trigeminal nerve (CN V) is most commonly observed.^[5,6]^ Consequently, various clinical symptoms, such as dizziness, tinnitus, hearing impairment, or facial pain can be present according to the involved nerve. Glossopharyngeal (CN IX) or vagal (CN X) involvement may cause dysphagia, hoarseness, or cardiac manifestation.^[7–9]^ There are few investigations of Ramsay Hunt syndrome involving upper cervical dermatome, but the mechanisms has not been clearly understood.^[10,11]^ Most of the affected patients had vesicular eruption involving cervical area as well as the auricle in the previous reports.^[10,11]^

In the current case, Ramsay Hunt syndrome with the cervical nerve involvement is uncertain due to the absence of vesicular formation in the cervical dermatomal distribution. However, it might be evidenced by the successful therapeutic effect of PRF to the great auricular nerve for the otalgia. Great auricular nerve originates from primary ventral ramus of the second and third cervical nerve, and it provides cutaneous branches to ear, external auditory canal, mandibular angle, and skin overlying a portion of the parotid gland.^[12]^ Indeed, Sweeney and Gilden^[2]^ reported that the presence of skin lesion was not essential for the diagnosis of Ramsay Hunt syndrome, which was understood as “zoster sine herpete”.^[13]^

Compared to the herpes zoster infection involving a more prevalent thoracic or cervical area, the outstanding symptom of Ramsay Hunt syndrome is motor weakness, the facial palsy. Therefore, treatment goals generally focus on the recovery of facial muscle function. Although standardized treatment guidelines for Ramsay Hunt syndrome has not fully been established, conservative treatment, including antiviral medication, corticosteroids, and analgesics are useful to relieve symptoms and to prevent undesirable sequela.^[14–16]^ The combination therapy (antiviral agent + corticosteroid) is known to fulfill successful recovery of facial palsy in most affected patients in several months.^[3]^ In this case, the facial palsy recovered dramatically with conservative treatment (antiviral agent + corticosteroid) in 2 weeks after the onset, but the patient had severe and persistent otalgia, that is, PHN following Ramsay Hunt syndrome, for more than 3 months. There have been few investigations regarding the development of PHN after Ramsay Hunt syndrome, despite it occasionally occurs in clinical practice.^[17]^

Interventional pain management such as nerve blocks on sympathetic and peripheral nerve or dorsal root ganglion (DRG) has been accepted as effective therapeutic options for herpes zoster and PHN.^[18,19]^ However, the appropriate intervention for chronic otalgia after Ramsay Hunt syndrome has not been clearly determined. Jacques et al^[17]^ reported the therapeutic effect of retroauricular infiltration, which was the landmark-based facial nerve terminal branch block, for chronic otalgia complicating Ramsay Hunt syndrome. The facial nerve contains only facial motor and special visceral efferent component in this extracranial distal portion.^[1]^ Therefore, the authors think that it was not the application for the original focus of otalgia. Nevertheless, we also experienced the positive therapeutic effect of ultrasound-guided facial nerve block for the patient, although transient. We suggest that the drug injected around the stylomastoid foramen could spread proximally along the facial nerve path and might reach the geniculate ganglion or nervus intermedius. The geniculate ganglion is the original site of viral reactivation and nervus intermedius is the sensory and parasympathetic division of the facial nerve.^[20]^ In this case, the authors also focused on the possibility of cervical nerve involvement for the development of chronic otalgia after Ramsay Hunt syndrome. PRF on the great auricular nerve block showed more long-lasting and potent analgesic effect, although both facial nerve block and great auricular nerve block caused transient symptomatic relief.

PRF is a safe and effective procedure for refractory neuropathic pain, and its efficacy has been proven in the treatment of PHN.^[21,22]^ The therapeutic mechanism of PRF includes temporal blockage of nerve signals and enhancement of the descending inhibitory pathway, although the exact mechanism is unclear.^[22,23]^ Changes in gene expression may contribute to long-term efficacy.^[23]^ Although PRF on the affected DRG or cranial nerve ganglion is a traditional technique, application on a peripheral nerve is also a useful alternative.^[24–27]^ For example, PRF on the intercostal nerve as well as on the DRG may be beneficial for patient with PHN of thoracic dermatome.^[24]^ Several researches demonstrated that PRF application to peripheral nerve or DRG caused neuronal change and pain regulatory gene expression in the proximity, that is, spinal cord.^[23,28]^ To the best of our knowledge, this is the first report of a patient with Ramsay Hunt syndrome followed by PHN, which was successfully treated with PRF to the great auricular nerve. The interventions to the great auricular nerve could be considered as the therapeutic options for refractory otalgia following Ramsay Hunt syndrome.

## 4 Conclusion

Chronic otalgia might be a type of PHN after Ramsay Hunt syndrome with cervical nerve involvement despite the absence of skin lesions in the cervical area. PRF treatment to the great auricular nerve can be a therapeutic option for refractory otalgia following Ramsay Hunt syndrome.

## Author contributions

**Data curation:** Hyungseok Lee.

**Methodology:** Ji-Seon Son.

**Writing – original draft:** Ye Sull Kim.

**Writing – review & editing:** A. Ram Doo.
